# Intrinsic Respiratory Gating for Simultaneous Multi-Mouse μCT Imaging to Assess Liver Tumors

**DOI:** 10.3389/fmed.2022.878966

**Published:** 2022-07-06

**Authors:** Mirko Thamm, Stefanie Rosenhain, Kevin Leonardic, Andreas Höfter, Fabian Kiessling, Franz Osl, Thomas Pöschinger, Felix Gremse

**Affiliations:** ^1^Experimental Molecular Imaging, RWTH Aachen University, Aachen, Germany; ^2^Gremse-IT GmbH, Aachen, Germany; ^3^Fraunhofer Institute for Digital Medicine MEVIS, Bremen, Germany; ^4^Discovery Pharmacology, Pharma Research and Early Development (pRED), Roche Innovation Center Munich, Penzberg, Germany

**Keywords:** respiratory gating, μCT, mice, liver tumor, hepatocellular carcinoma, iterative reconstruction, small animal, high through put

## Abstract

Small animal micro computed tomography (μCT) is an important tool in cancer research and is used to quantify liver and lung tumors. A type of cancer that is intensively investigated with μCT is hepatocellular carcinoma (HCC). μCT scans acquire projections from different angles of the gantry which rotates X-ray source and detector around the animal. Motion of the animal causes inconsistencies between the projections which lead to artifacts in the resulting image. This is problematic in HCC research, where respiratory motion affects the image quality by causing hypodense intensity at the liver edge and smearing out small structures such as tumors. Dealing with respiratory motion is particularly difficult in a high throughput setting when multiple mice are scanned together and projection removal by retrospective respiratory gating may compromise image quality and dose efficiency. In mice, inhalation anesthesia leads to a regular respiration with short gasps and long phases of negligible motion. Using this effect and an iterative reconstruction which can cope with missing angles, we discard the relatively few projections in which the gasping motion occurs. Moreover, since gated acquisition, i.e., acquiring multiple projections from a single gantry angle is not a requirement, this method can be applied to existing scans. We applied our method in a high throughput setting in which four mice with HCC tumors were scanned simultaneously in a multi-mouse bed. To establish a ground truth, we manually selected projections with visible respiratory motion. Our automated intrinsic breathing projection selection achieved an accordance of 97% with manual selection. We reconstructed volumetric images and demonstrated that our intrinsic gating method significantly reduces the hypodense depiction at the cranial liver edge and improves the detectability of small tumors. Furthermore, we show that projection removal in a four mice scan discards only 7.5% more projections than in a single-mouse setting, i.e., four mouse scanning does not substantially compromise dose efficiency or image quality. To the best of our knowledge, no comparable method that combines multi-mouse scans for high throughput, intrinsic respiratory gating, and an available iterative reconstruction has been described for liver tumor imaging before.

## Introduction

Small animal micro computed tomography (μCT) is an important tool in cancer research. Since the animals can be examined non-invasively and repeatedly, the number of animals to be sacrificed in a longitudinal study can be drastically decreased. μCT is also used in combination with single photon emission computed tomography (SPECT) and positron emission tomography (PET) to provide anatomical information. However, standalone μCT is also widely used due to its fast acquisition time, high spatial resolution resulting in accurate anatomical information, relatively simple image analysis, and diverse application possibilities (1–3).

A type of cancer that is intensively investigated with μCT is hepatocellular carcinoma (HCC). Now-a-days, it is the prevailing liver cancer with increasing incidence (4). Multiple HCC mouse models are used preclinically to assess the treatment response of new drugs (1). For example, Hage et al. (1) characterized two HCC models, investigated their response to immune checkpoint blockers (anti-PD-1and anti-CTLA-4), and determined the orthotopic tumor growth by *in vivo* μCT imaging. For the assessment of liver tumors, two main issues need to be addressed particularly: soft tissue contrast and high throughput scanning to reduce the necessary time and costs.

μCT image contrast is based on the absorption of X-rays and is therefore highly suitable to examine radiodense structures like bones, with high resolution down to the sub-micrometer range (5). However, it often provides insufficient soft tissue contrast, which is needed to investigate liver tissue and liver tumors (6). To overcome this problem, a radiodense contrast agent is used to increase contrast between soft tissues of interest. Because of the longer acquisition times of preclinical μCTs and the fast pharmacokinetics of clinical contrast agents, these clinical approaches cannot be simply transferred to preclinical research. Additionally, toxicological side-effects of preclinical contrast agents have to be considered (3). However, liver tumors and metastases can be assessed and monitored over time using contrast agents such as ExiTron nano, eXIA, or Fenestra, which accumulate in healthy liver tissue and increase the radiodensity compared to tumor tissue (1, 7, 8).

A μCT scan acquires projections from equidistant angles by rotating the X-ray source and detector around the sample. Afterwards, a reconstruction algorithm computes a 3D volume from these projections. Motion inside the sample causes inconsistencies between projections which leads to artifacts in the resulting image. Thereby, motion by the heartbeat and breathing cause artifacts while scanning anesthetized animals. Scan augmentations known as cardiac and respiratory gating can reduce the problematic motion effects and improve the image quality. This can help to visualize certain pathologies that would otherwise be invisible (9). Moreover, measurements of tumors are more exact with gating, because it reduces the blurring at the edge of pathological structures (2, 9). For extrinsic gating, a motion signal is obtained by an external hardware like a ventilator for forced ventilation (10, 11) or a respiratory pad (12) for non-ventilated mice. For intrinsic gating [synonymous with self-gating, image-based, or raw-data-based gating (13)], the motion is calculated from the projection data itself (9, 14). An extrinsic motion signal can be applied prospectively or retrospectively (9, 15). In prospective gating, the X-ray exposure is actuated by the sought-after phase of the physiological motion (16, 17). This strategy avoids reconstruction artifacts, because it provides uniform angular projections but needs exceedingly long scan times (~10 min) (15) and requires complicated hardware like a pulse-able radiation source or a quick shutter mechanism, which is usually not available for μCT. In retrospective gating, the motion affected projections are detected after the scan. That can also be done extrinsically by applying a recorded external signal (18) or intrinsically (14). To avoid missing angles, the acquisition protocol usually requires multiple projections for every gantry angle (19) because the reconstruction would otherwise suffer from artifacts (15). However, this again leads to longer scan times and potentially higher X-ray exposure. The dose should be as low as possible, because radiation can affect the immune system and other biological pathways of the animals, particularly in longitudinal imaging studies (20). To avoid both a high radiation dosage and long scan times, iterative reconstructions were suggested to minimize artifacts (2, 21, 22).

Our approach uses a model-based iterative reconstruction. Whereas this type of reconstruction is computationally intensive, it allows projections to be discarded without substitution. This is an important distinction to some previously described methods where retrospective phase binning or interpolation (13, 23) is used to re-create equidistant projections.

**Figure 1:**
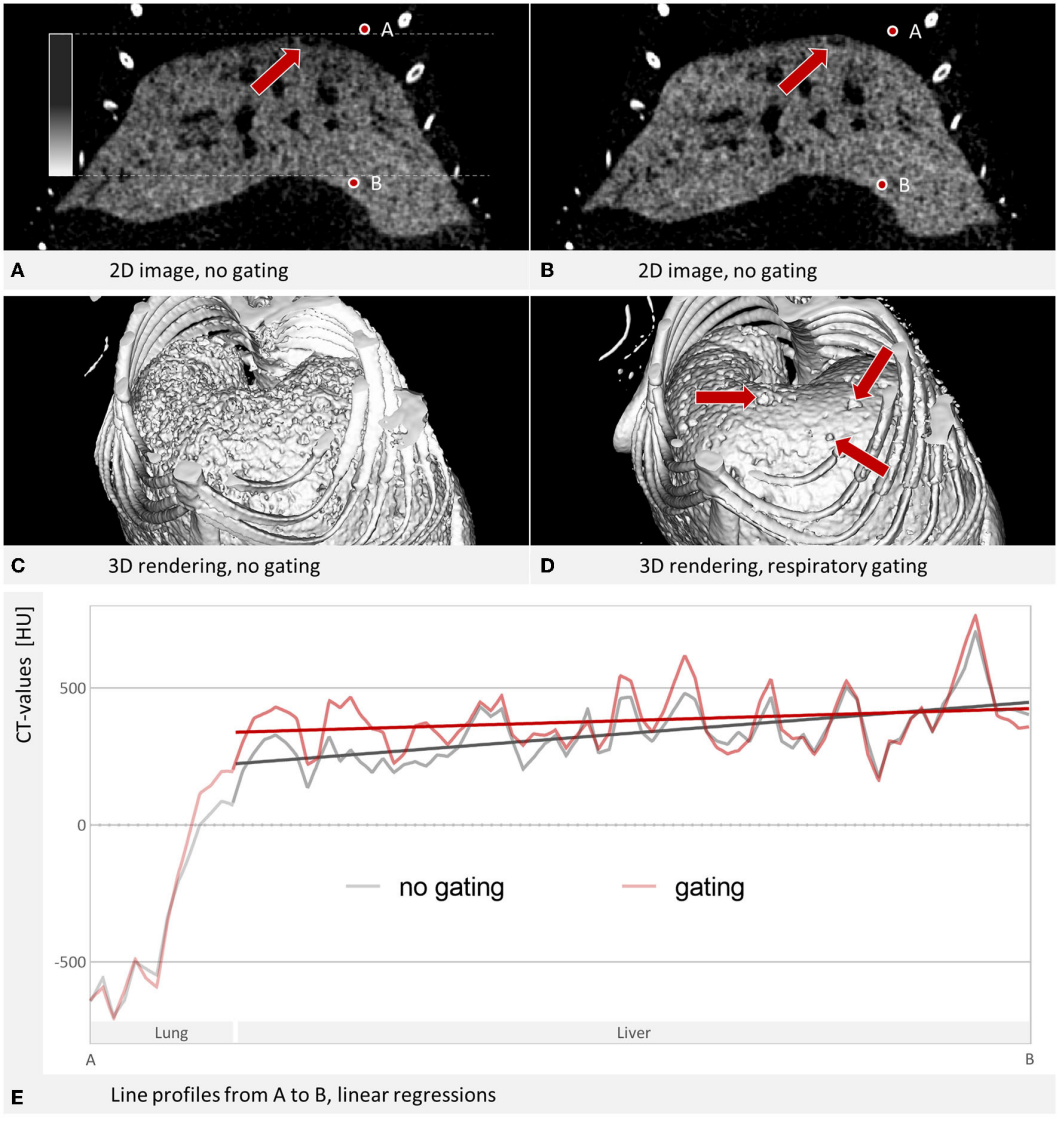
Liver tumor imaging. **(A)** Coronal slice through a contrast-enhanced mouse liver, reconstructed without gating. W: 1,007 HU L: 443 HU. Tumors and blood vessels appear as dark regions. A hypodense depiction looking like a gradient at the cranial edge of the liver occurs due to contributions from the lung. The tumor marked by the arrow is hardly visible. **(B)** Respiratory gating improves contrast at the liver edge and increases the detectability of the marked tumor. W: 1,007 HU L: 443 HU. **(C)** The liver shows a rough/uneven surface in the 3D rendering. **(D)** The 3D rendering of a gated reconstruction shows a smoother surface with detectable tumors. **(C+D)** Are simple surface rendering done by the commercially available software mentioned under materials and methods. **(E)** Line profile from point A to point B, covering 6.96 mm. Linear regressions over the segment that covers the liver show a steeper slope without gating (37.5 HU/mm) than with gating (14.6 HU/mm).

For *in vivo* studies that target liver tissue and liver tumors, it is important to consider the respiratory motion, because the region of interest moves with every breath due to anatomical proximity to the lung. Therefore, liver imaging suffers more from breathing artifacts than imaging of subcutaneous tumors, for example. *In vivo* imaging of mice requires special strategies because of their fast physiological motion (16). One major effect of the respiratory motion is a blurring at the cranial edge of the liver. It often looks like a gradient with hypodense intensity toward the diaphragm which makes it harder to evaluate HCC tumors in that region, see Figures 1A,B. To counteract these motion artifacts, respiratory gating can be applied for improved liver imaging.

Inhalation anesthesia (e.g., isoflurane) leads to a regular respiration with short phases of gasping breathing and long phases with barely any motion (9, 24). This makes inhalation anesthesia ideally suited for intrinsic retrospective gating (9). Usage of an iterative reconstruction allows for basically any μCT scan to discard the relatively few projections in which the gasping motion occurs and therefore still have sufficient data for the reconstruction. That is the basis for the approach of this study.

An important parameter for our gating approach is the *rejection fraction* (RF), i.e., the fraction of projections which should not be included in the reconstruction. This can roughly be regarded as the sensitivity of the motion detection algorithm. The following effects are expected to occur with an increasing RF, and not all are improving the image quality:

1) The contribution of the lung to the liver edge region is reduced, increasing signal in the liver and contrast to the lung, decreasing the gradient toward hypodense intensities at the cranial edge of the liver.
2) The remaining projections are more consistent leading to less streaking artifacts.
3) Less projections mean less raw data, which leads to more noise.

**Figure 2:**
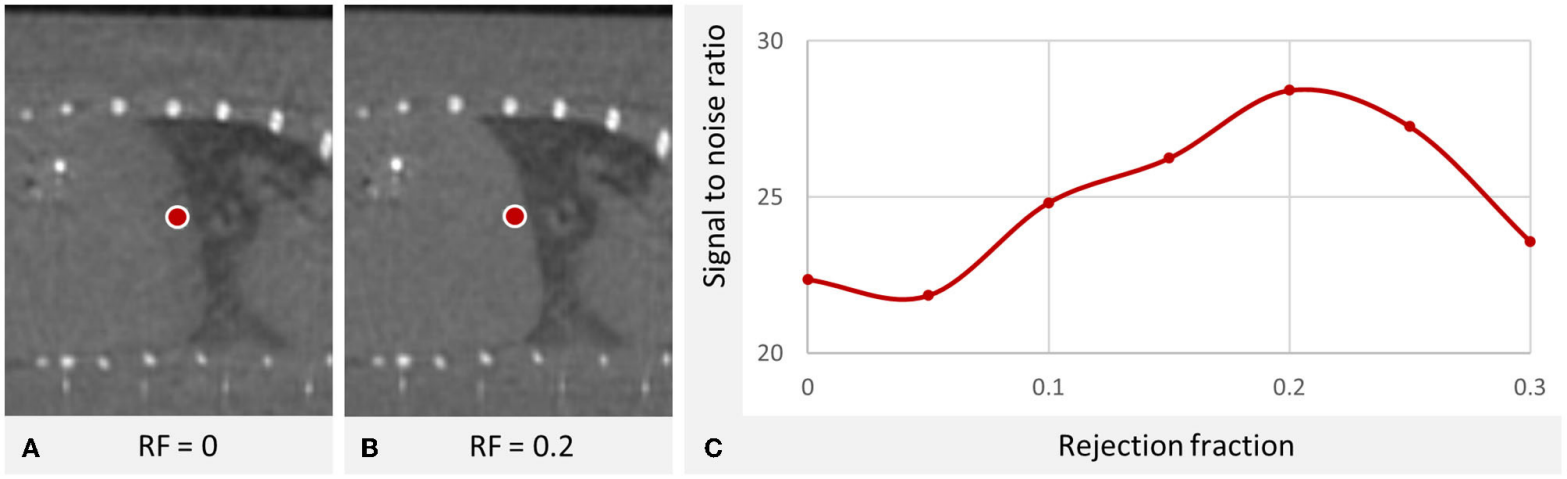
Optimal rejection fraction in a μCT scan of the liver of a single mouse without contrast agent. **(A** vs. **B)** The breathing of the mouse causes a hypodense depiction of the cranial liver parts which is reduced using respiratory gating. W: 2,487 HU L: 50 HU. **(C)** The signal was measured at the marked cranial liver region for different rejection fractions (RF) showing an optimum at RF=0.2. (μCT device U-CT, MILabs B.V., Utrecht, the Netherlands, continuous rotation mode, tube voltage of 65 kV exposure time 75 ms, tube current 0.13 mA). The optimum is a compromise for which inconsistent projections are removed with the trade-off that less data is available for reconstruction.

Therefore, an optimal rejection fraction needs to be experimentally determined. To illustrate this problem, Figure 2 shows a μCT scan of a single mouse without contrast agent. A rejection fraction of 0.2 improves the image quality and signal-to-noise ratio at the liver edge, but higher rejection fractions are suboptimal again.

A higher throughput can be accomplished by scanning multiple mice simultaneously using special mouse holders. As already described before, some devices can hold two (25) or four (26, 27) mice. High throughput is a conflicting goal with high image quality though, because multiple mice also multiply the overall physiological motion brought into the scan by each animal. Multiple respiratory pads can be used with a multi-mouse bed, but they take up valuable space inside the μCT and do not provide the information about motion contributions across mice, as described below. Additionally, the required external hardware is expensive, error prone, and requires time for setup before scanning.

For a singlemouse setting, retrospective intrinsic gating in combination with an iterative reconstruction has been used before and proven to improve tumor volume accuracy compared to non-gated images (2). However, to the best of our knowledge, no comparable method that combines multi-mouse scans for high throughput, intrinsic respiratory gating and a commercially available iterative model-based reconstruction has been described before and evaluated for liver tumor imaging. We present and test a method that can be used for multi-mouse scans to assess the liver tumor state in high throughput settings and does not require any additional measuring hardware such as breathing pads. We show that a retrospective intrinsic gating of a four-mice scan leads to the rejection of only 7.5% more projections compared to a single-mouse setting. Our model-based iterative reconstruction eliminates the need to acquire multiple projections at each angle and can create an image of competitive quality. Moreover, this method can be applied to basically all existing μCT scans.

## Materials and Methods

The raw data basis for this study consists of three scans. For each scan four mice with liver tumors were placed in a multi-mouse bed and thus, scanned simultaneously. We examined each projection of each scan to manually select breathing projections and establish a ground truth for evaluation of our automated breathing projection algorithm. Reconstructions were then performed for different rejection fractions to assess the effect on image quality.

### Reconstruction

Volumetric images were reconstructed using our μCT reconstruction software on a PC with Microsoft Windows 10 equipped with an Intel Core i7-5930K processor, 32GB RAM, and an NVIDIA RTX 2080 Super GPU. The reconstruction software is commercially available (CTRecon, Gremse-IT GmbH, Aachen, Germany) and offers a model-based iterative reconstruction. Since iterative reconstruction is much more computationally intensive than direct reconstruction, our iterative reconstruction software makes use of multi-GPU-acceleration, if available, and requires only a few minutes of reconstruction time per mouse when reconstructing at typical resolutions around 80 μm.

**Figure 3:**
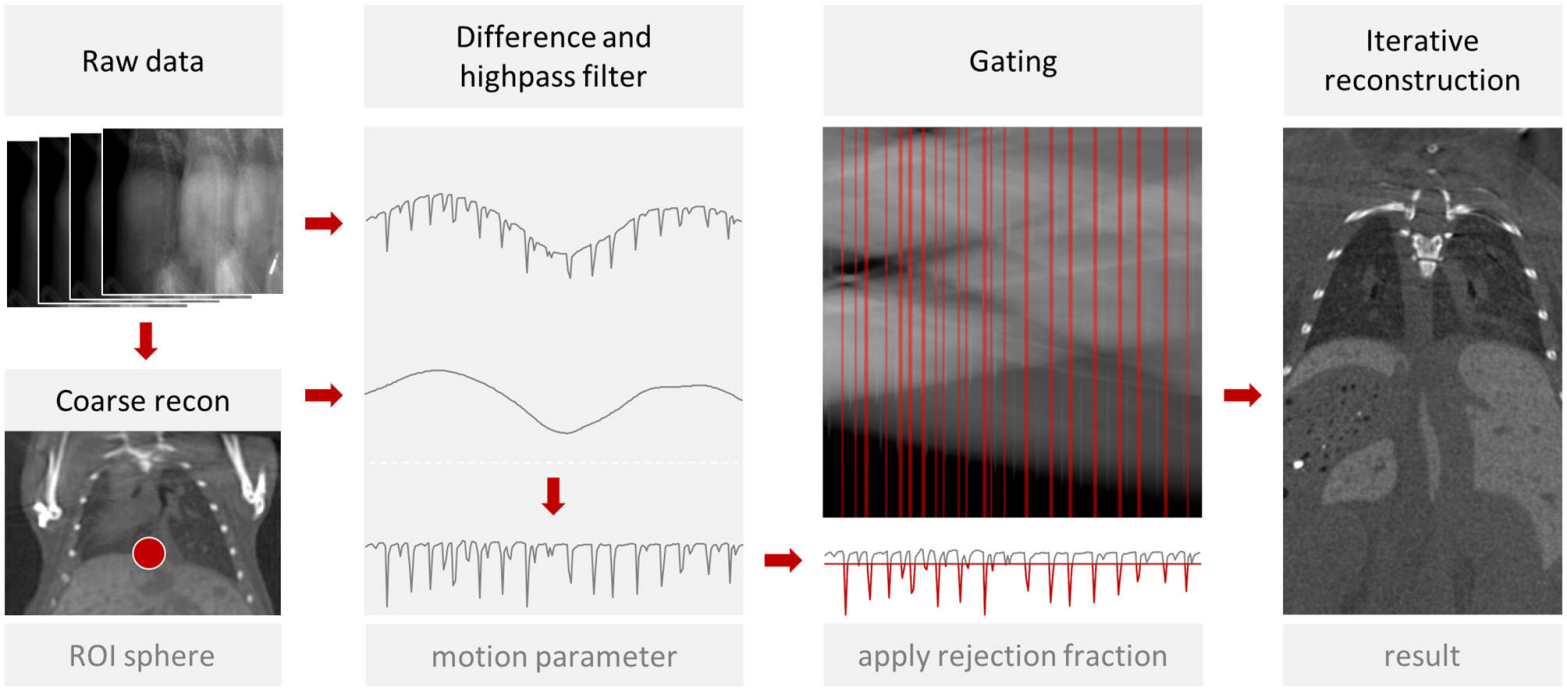
Gated reconstruction approach. A coarse preview is reconstructed from the raw data and the user can mark the diaphragm with a sphere (ROI). From the signal intensity inside the projected sphere, the algorithm computes a motion score for each projection and rejects a fraction of the projections. From the remaining projections, a reconstruction is computed. This process is repeated for each mouse of a multi-mouse scan.

**Figure 4:**
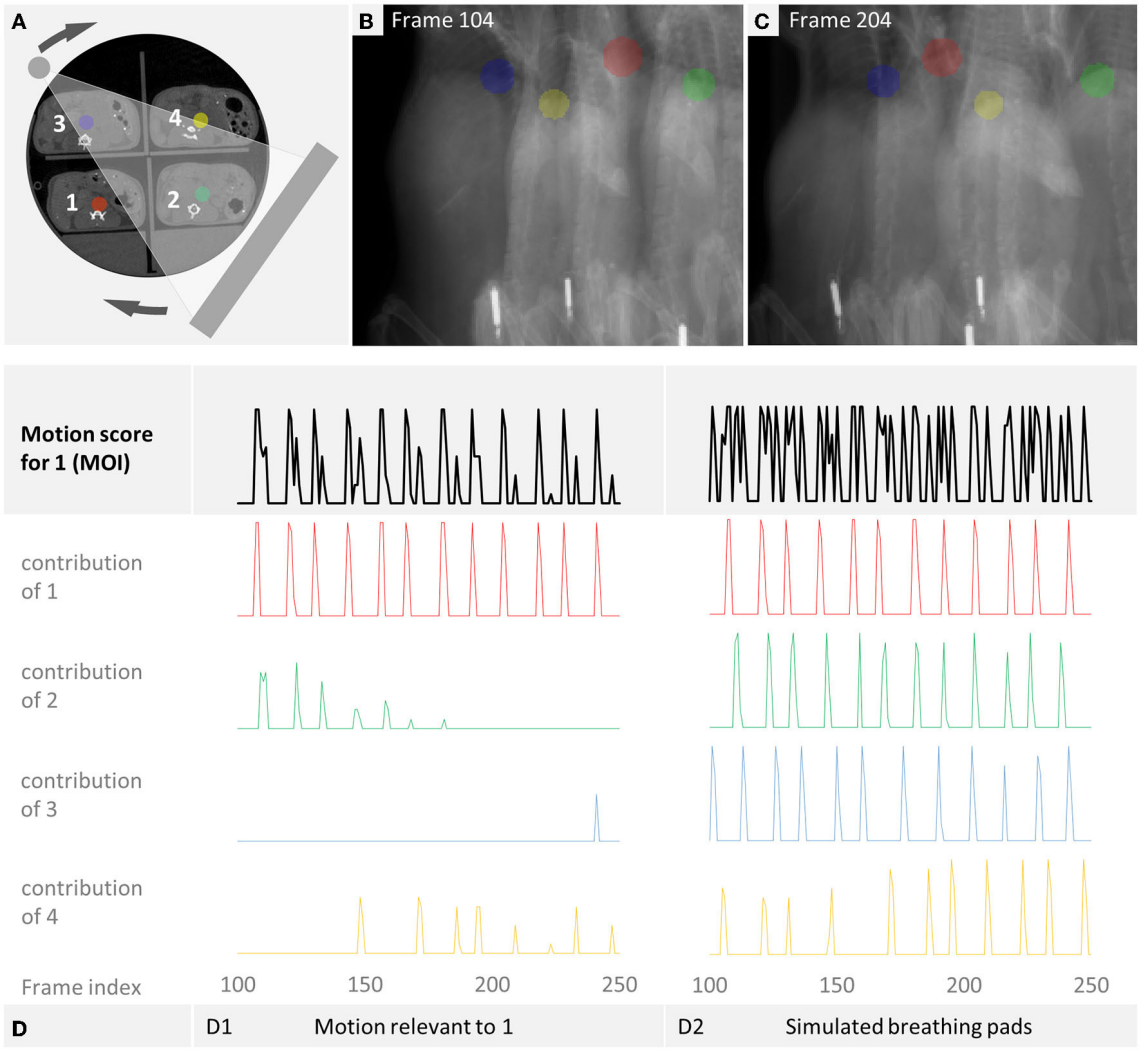
Overlap of respiratory motion. We determined the motion score for mouse 1 (MOI). All curves are derived from the manual breathing examination of all projections. **(A)** The x-ray source and detector rotate around the sampled multi-mouse bed. **(B,C)** Show the different resulting projections. **(D1)** Shows the motion score for a simulated 4-mice-scan with overlap-based projection rejection that results when only those respiratory motions are considered which overlap the MOI. **(D2)** Simulates a 4-mice scan with breathing pads. Lacking the information, in which mice overlap in which projection, every respiratory motion needs to be considered. The resulting cumulative curve of the motion score shows irrelevant contribution of other mice leading to unnecessary projection rejection.

The processing steps of the gating are described in Figure 3. The software first computes a coarse preview reconstruction using the raw data, requiring only a few seconds. Then, the user can place a sphere forming a region of interest (ROI) at the diaphragm of the mouse of interest (MOI). User interactions to place a ROI have been described before (13, 14, 28). The reconstruction software projects the sphere onto each acquired projection and computes the mean signal intensity inside the projected sphere to calculate a curve. The projected sphere intensity of the coarse reconstruction is subtracted to remove the changes that result from the rotation. Then, for low pass filtering, a median filter with radius 4 is used, resulting in a smoothed curve. This curve is then subtracted to achieve a high-pass filter. Finally, a threshold is determined and applied to reject a number of breathing projections according to the rejection fraction. This is similar to previous approaches (28). However, in our case with the 4 mice, the reconstruction of the image of the MOI can also be affected by motion that is caused by other mice behind or in front of the MOI. That does not apply to every breathing cycle though. Only those projections are affected in which the breathing mouse and the MOI overlap, as it is visualized in Figure 4. To clarify, even if four mice are scanned at once, a specific reconstruction is performed for each individual mouse. Each reconstruction is performed using a selection of projections specific to that mouse. The above-mentioned threshold is defined by the *rejection fraction* (RF) which indicates the fraction of the projections omitted from the reconstruction. At a rejection of 0.2, for example, 20% of projections in which the motion parameter has the lowest values are rejected.

The iterative reconstruction itself involves a forward model which computes the projections using line integrals which are implemented using GPU-accelerated texture-based trilinear filtering. A beam hardening model is applied to account for x-ray source distribution, tissue absorption and detector response curve. For this study we apply a Gaussian smoothing (1.34 stddev) of the measured projections followed by 3x3 software binning to achieve an appropriate balance between noise and resolution (~150 μm) for this application. The weighted squared difference between simulated and measured pixels is used as cost term where the pixel weight is based on the pixel intensity assuming a poisson-like noise model. To minimize the cost term, the linear conjugate gradient method is used with 30 iterations where the gradient is computed using GPU-accelerated adjoint algorithmic differentiation (29).

We reconstructed volumes of each mouse with rejection fractions from 0 to 0.6 in steps of 0.05. Therefore, our data consists of 156 (= 3 scans times 4 mice per scan times 13 rejection fractions) volumetric images in total. Each volume was reconstructed with a voxel size of 80 μm. The reconstruction dimensions are 777x442x752 (scan 1), 800x427x800 (scan 2) and 800x465x800 (scan 3).

### Image Analysis

Images were visualized and analyzed using the software Imalytics Preclinical 3.0 (Gremse-IT GmbH, Aachen, Germany) (30).

To form a ground truth for further analysis, we manually examined each projection of every scan for each mouse and compared it to the preceding and following projections. In this way, we could visually detect respiratory motion and determine whether it affects the MOI. During the manual review, we decided for each projection how much the possible respiratory motion of each mouse affects the MOI. We noted this in integral numbers from 0–10. A clear full motion of the diaphragm of the MOI itself corresponds to 10, as does a corresponding motion of another mouse that overlaps with the MOI in the projection under consideration. We have devalued a weaker motion or a very slight overlap accordingly. If there is no overlap with the MOI, a breathing motion is irrelevant and was noted with 0. To simulate gating, which can only accept or discard projections, we defined the following threshold: Projections with a motion value ≥ 3 are discarded.

For quantitative inspection of the image quality, we set the following measurement points or areas in each volume:

- Start- and endpoint for a line profile over the liver starting at the cranial edge (see **Figure 7**).
- 3 spherical regions of 1 mm diameter at the cranial edge of the liver (see **Figure 8**).
- 3 spherical regions of 1 mm diameter in a central area of the liver but outside tumors (see **Figure 8**).
- 3 segmentations of liver tumors (see **Figure 9**).
- 1 segmentation of a homogenous area of the mouse bed.

### Statistical Analysis

Comparisons between two groups were performed using the Student's *t*-test. For a comparison of more than two groups, statistical analysis was performed by one-way analysis of variance (ANOVA). Statistical significance of *p* < 0.001 is indicated ^***^ and *p* < 0.0001 is indicated ^****^. For statistics, linear regression and graphs the software GraphPad Prism (version 9.2.0, GraphPad Software Inc., San Diego, CA, USA) was used. The line charts were rendered with Microsoft Excel 365 (Redmond, WA, USA).

### Micro-Computed Tomography (μCT) Imaging

Image acquisition was performed using a micro-computed tomography device (U-CT, MILabs B.V., Utrecht, the Netherlands). Anesthetized mice were placed in a commercially available multi-mouse bed (MILabs B.V., Utrecht, the Netherlands) and a scan of liver and lungs was performed. In a full-rotation in step-and-shoot mode, 1440 projections (1944 x1536 pixels) were acquired with an X-ray tube voltage of 55 kV, tube current 0.17 mA, exposure time of 75 ms, source isocenter distance of 117.578 mm, and source detector distance of 297.459 mm. The beam was hardened by the filters: Aluminum 100 μm (fixed filter) + aluminum 400 μm (from a filter wheel). For each scan a dose of 344 mGy was applied according to the manufacturers information which was assessed for our scan protocol using a phantom.

To induce anesthesia, 4% isoflurane in oxygen-enriched air using a dedicated vaporizer was applied to the animals. During imaging, the isoflurane concentration was reduced to 2.0% and maintained on this level. This inhalation anesthesia caused the above-mentioned regular respiration with short gasps and long phases of nearly no motion. Eyes were protected from dehydration with Bepanthen eye ointment.

Liver contrast was enhanced by intravenous injecting of 100 μl of ExiTron nano 6000 (Viscover, Miltenyi Biotec, Bergisch Gladbach, Germany) at least 4 h before first image acquisition. This contrast agent causes a long-lasting enhancement of healthy liver tissue, allowing to track tumor which appear as hypodense structures over months.

### Mice and Cell Lines

All animal experiments were approved and conducted according to the regulations from the Government of Upper Bavaria (Regierung von Oberbayern; Approval Number: ROB55.2-2532.Vet_03-15-89) and performed in accordance with the Federation for Laboratory Animal Science Associations (FELASA). The animal facility has been accredited by the Association for Assessment and Accreditation of Laboratory Animal Care (AAALAC).

Female mice (iAST transgenic mice) were obtained from Charles River Laboratories (Sulzfeld, Germany).

For the generation of the orthotopic iAST mouse model, 5x10^8^ infectious units (IU) of adenovirus (Ad.Cre) expressing Cre recombinase (Vector BioLabs, Malvern, PA, USA) were injected intravenously into 6–8 week old inducible AST (iAST) mice as described in Stahl et al. (31). The iAST mice express the SV40 large T antigen with a hepatocyte-specific albumin promoter. Conditional expression is regulated by a loxP flanked stop cassette. After the experiments, mice were euthanized by cervical dislocation. A body weight loss of 20% and more was defined as an endpoint criterion.

## Results

### Effect of Overlapping Mice on Projection Rejection

A μCT scan acquires projections from many, often equidistant, angles. Figure 4A illustrates how the rotating X-ray source and detector result in different projections (B–C). We manually examined each projection of every scan for each mouse respectively to detect respiratory motion. We used the manual labeling to *simulate* the projection selection for the following cases:

1. **Single-mouse scan**. Only the respiratory motion of the MOI itself is considered. Therefore, the same projections are rejected as for a single-mouse scan.
2. **4-mice scan with overlap-based projection rejection**. When the projection of the MOI overlaps the projection of another mouse, the respiratory motion of the other mouse should be considered for gating, leading to projection rejections for the MOI. On the other hand, respiratory motion of other mice which do not overlap can be ignored as far as the MOI is concerned. Our algorithmic approach aims to allow for an optimized gating leading to a 4-mice scan with overlap-based projection rejection. This is shown in Figure 4D1. The motion parameter for the MOI (black) has only slightly more deflections than the motion curve of the MOI itself (red). The remaining curves show the breathing motion of the other mice, as far as they are relevant for the MOI, i.e., they happen in an overlapping projection.
3. **4-mice scan with breathing pads**. A standard setup with respiratory pads misses the information which mice overlap in which projections. Therefore, a conservative gating would discard every projection in which at least one mouse shows respiratory motion. Figure 4D2 simulates a 4-mice scan with breathing pads. Every respiratory motion of each mouse is considered for the motion parameter which shows many deflections. This approach has the disadvantage that more data is discarded than necessary. Another possibility would be to only discard the projections in which the MOI breathes. However, this would still lead to artifacts when motion occurs in a mouse that at the time of this projection overlaps the MOI.

**Figure 5:**
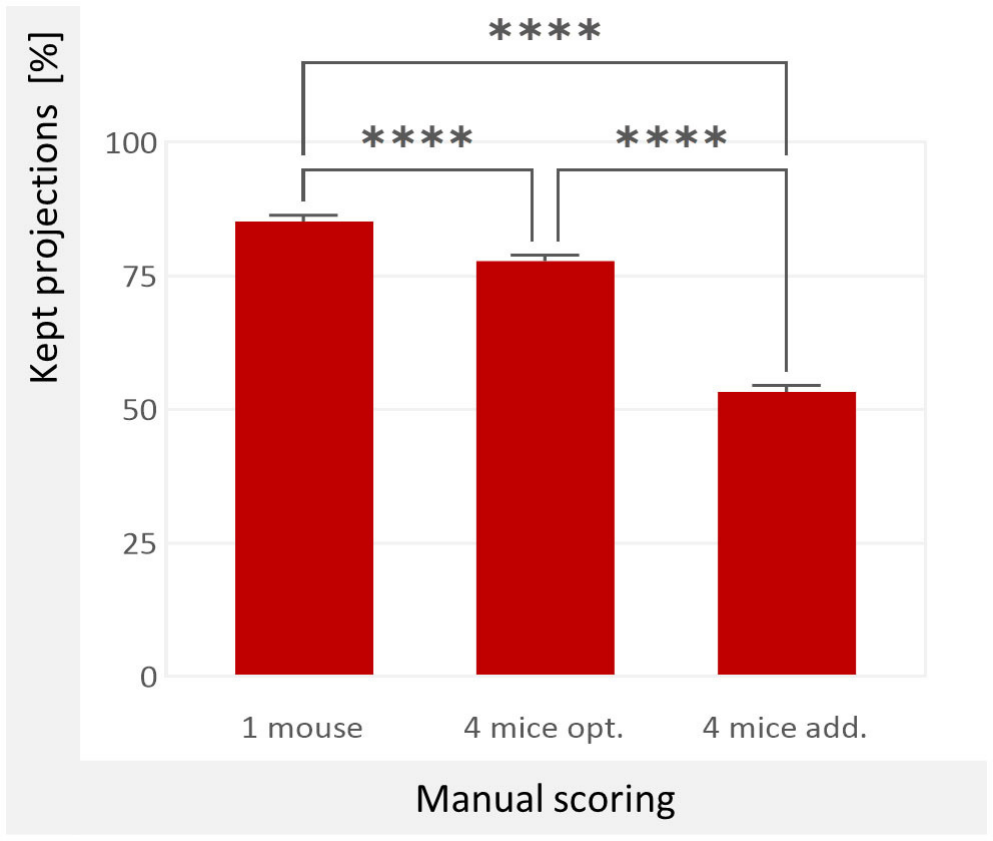
Used projections according to manual motion detection. We averaged the manual examination of all twelve mice. 1 mouse: 85.22 %; Only the MOI is considered, which emulates a single-mouse scan. 4 mice opt: 77.78 %; all mice but only their relevant motion is considered, which simulates a 4-mice-scan with overlap-based projection rejection. 4 mice add.: 53.38 %; each motion is added up without considering the actual overlap, which simulates a 4-mice scan with breathing pads and conservative projection selection. The values differ significantly *(p* < 0.0001 as indicated by ****, one-way ANOVA).

We compared how many projections can be included in the reconstruction averaged across all data sets. The results are shown in Figure 5. Particularly noteworthy is the small difference between the simulated s*ingle-mouse scan* (85.22%) and the *4-mice scan with overlap-based projection rejection* (77.78%). This difference is <7.5% (absolute, or 9.6% relative). If, on the other hand, a *4-mice scan with breathing pads* is simulated by considering the respiratory motion of all mice, only 53.38% of the projections could be used for the reconstruction. A noticeable difference that would greatly affect the image quality.

### Evaluation of Automated Projection Selection

**Figure 6:**
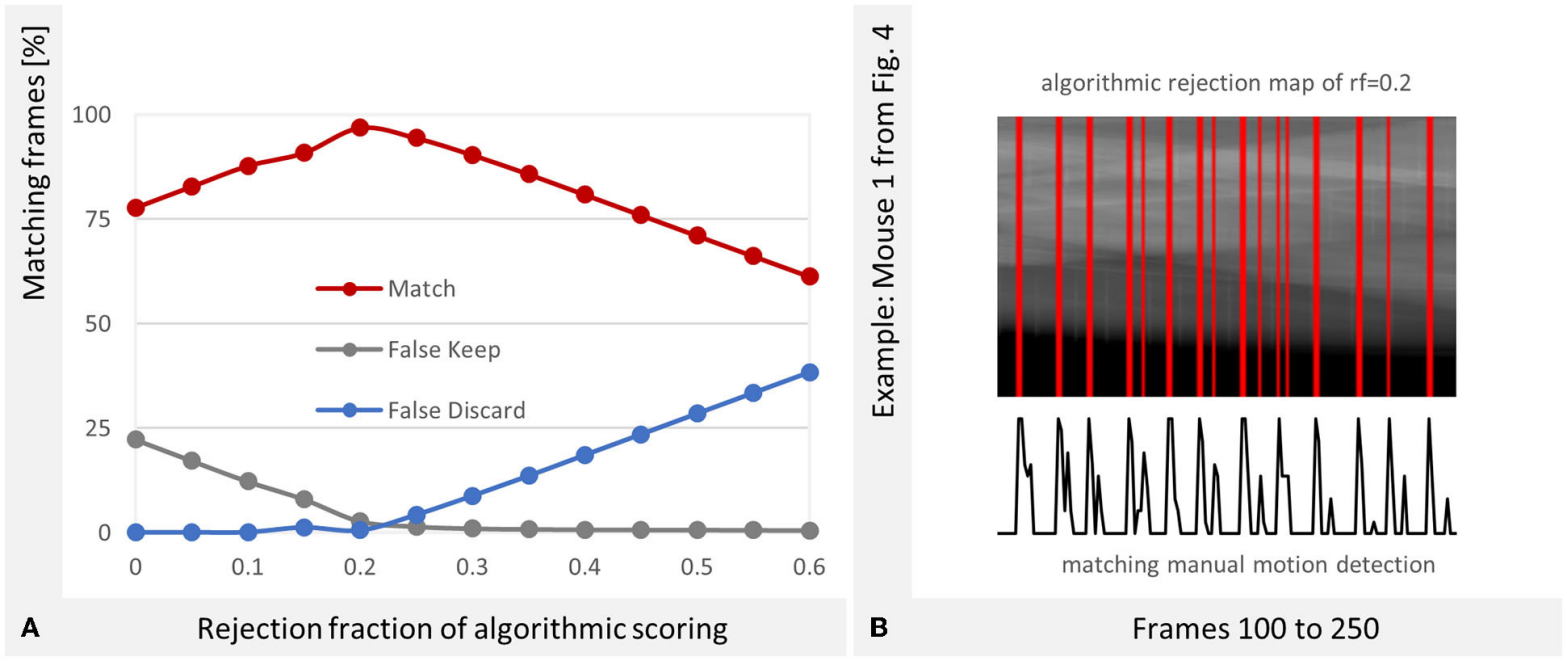
Optimal Rejection Fraction (RF). **(A)** The best correspondence between the manual evaluation and the algorithmic gating is at RF = 0.2. The success rate is 96.92%. **(B)** The reconstruction software provides a projection rejection map which is depicted as red stripes over the projection sinogram. The peaks of manually detected motion (see Figure 4B) match the algorithmically created stripes in the map.

The manual labeling of the motion in all projections allows an evaluation of the automated projection selection algorithm. The results are shown in Figure 6. The best match with the manually determined ground truth is in our use case a rejection fraction of 0.2. This coincides expectedly with the result in Figure 5.

The algorithm considers the ROI at the diaphragm of the MOI. In case of an overlapping motion, the signal intensityof the projection changes there as well which is recognized. We can state that the algorithm, which was originally designed for a single-mouse scan also detects respiratory motion in overlapping mice well.

### Image Intensity at Liver Edge and Center

**Figure 7:**
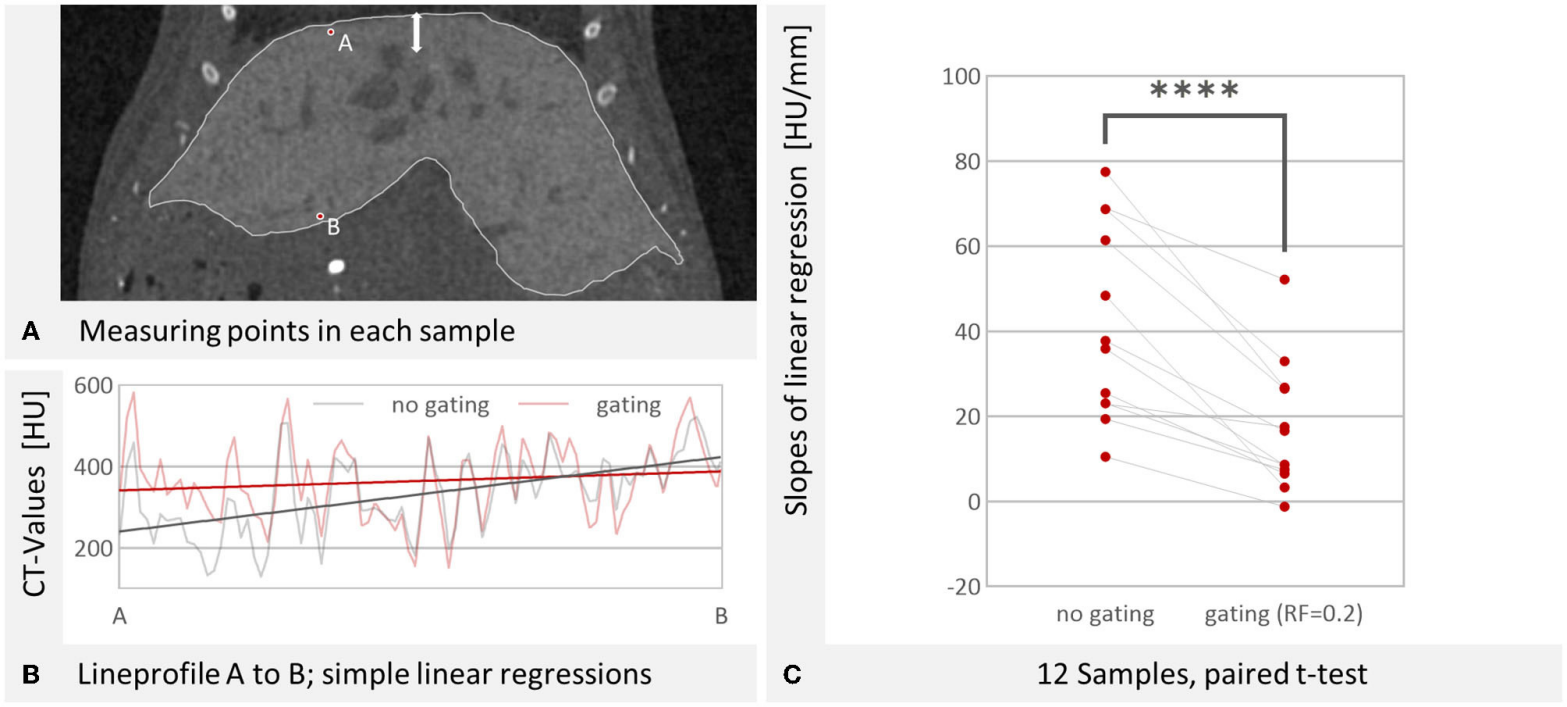
Evaluation of line profiles. **(A)** Start- and endpoint for the evaluated line profile. The breathing results in a motion of the lungs and liver as shown by the white arrow. Image reconstructed with RF = 0.2. W: 4,014 HU L: 668 HU **(B)** Lineprofile from point A to point B. Linear show a steeper slope without gating (25.5 HU/mm) than with gating (6.5 HU/mm). The lineprofile covers 7.2 mm. **(C)** The slope is significantly reduced by gating (for RF = 0.2, *n* = 12, *p* < 0.0001 as indicated by ****, paired *t*-test).

The gradient toward hypodense intensities at the cranial edge of the liver is a problem for liver imaging that can be addressed with respiratory gating. In slices, it appears as a gradient that becomes darker toward the diaphragm, in 3D images as a frayed surface (Figure 1). We also exemplified that linear regression over the line profile of the liver slopes in the cranial direction, which can be improved by gating. We investigated this for all samples (Figure 7) and could determine that the slope is significantly reduced with gating (RF = 0.2).

**Figure 8:**
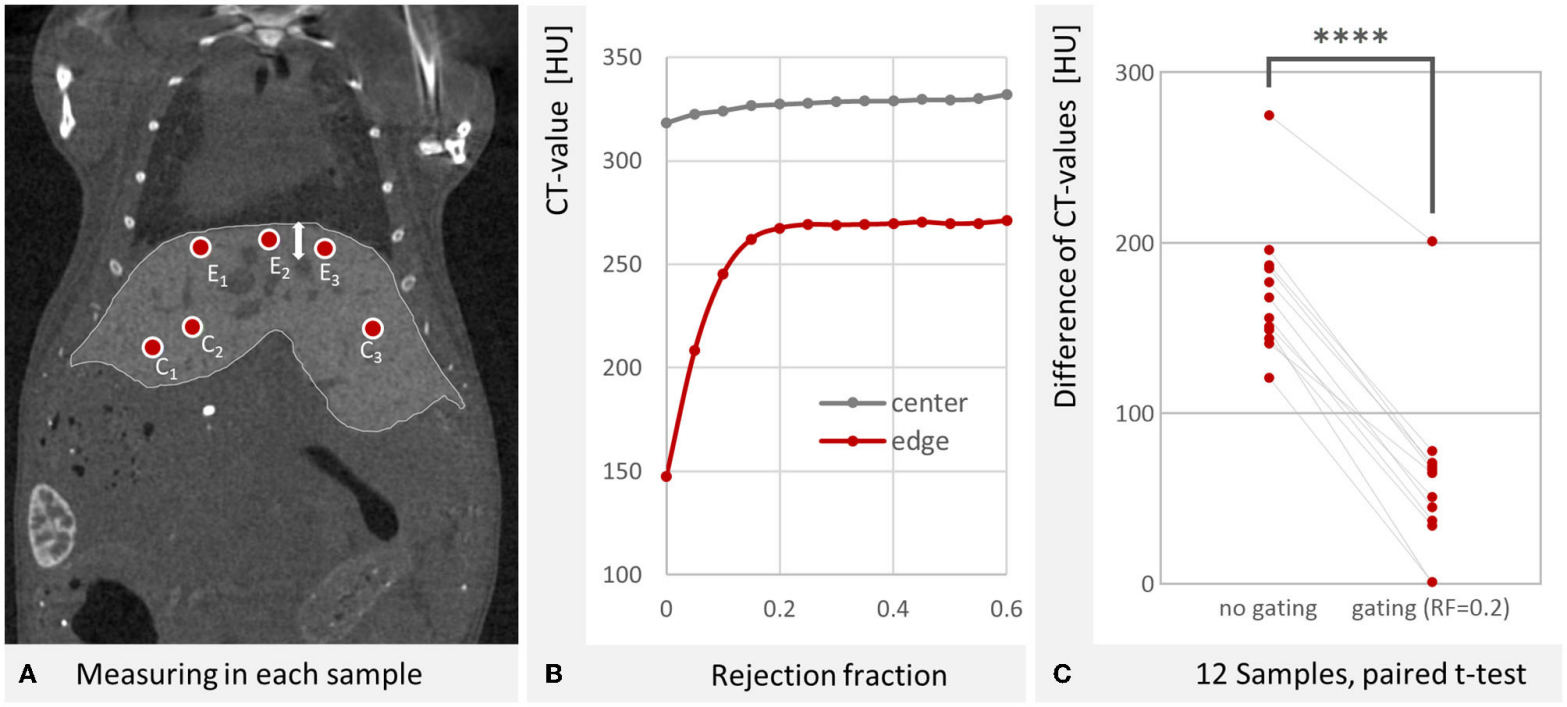
Gating reduces the hypodensity at the liver edge. **(A)** Evaluated regions at the center of the liver C_1−3_ and near the edge toward the diaphragm E_1−3_. The gasping breathing results in a motion of the lungs and liver as shown by the white arrow. At the cranial liver edge (E_1−3_), the contribution of motion projections causes a hypodense depiction. Image reconstructed with RF = 0.2. W: 4,014 HU L: 668 HU **(B)** The hypodense depiction at the liver edge is reduced with increasing rejection fractions. For RF > 0.2 no noticeable improvement is found. **(C)** The difference between liver center and edge CT-values is significantly reduced by gating (for RF = 0.2, *n* = 12, *p* < 0.0001 as indicated by ****, paired *t*-test).

Since the line profiles appeared to be quite noisy and were affected by blood vessels and tumors they passed through, we also assessed the effect with regions that we placed in apparently homogenous liver regions. We determined the difference between the mean CT-value of three spherical regions in the center of the liver and three spherical regions at the edge of the liver. These regions were placed for each mouse and the intensities were computed for a range of rejection fractions. This difference was found to be smaller for each mouse with gating (RF = 0.2) than without gating. This is illustrated in Figure 8 and we found that the hypodensity at the liver edge was significantly (*p* < 0.0001, paired *t*-test) reduced by gating. The effect increases with the rejection fraction, but no noticeable improvement occurs for values above 0.2 (Figure 8B). Even for high rejection fractions, the liver center remains somewhat more radiodense, however. We do not know whether this is a result of the contrast agent distribution, vascularization, or a remaining breathing motion artifact.

### Detectability of Tumors

**Figure 9:**
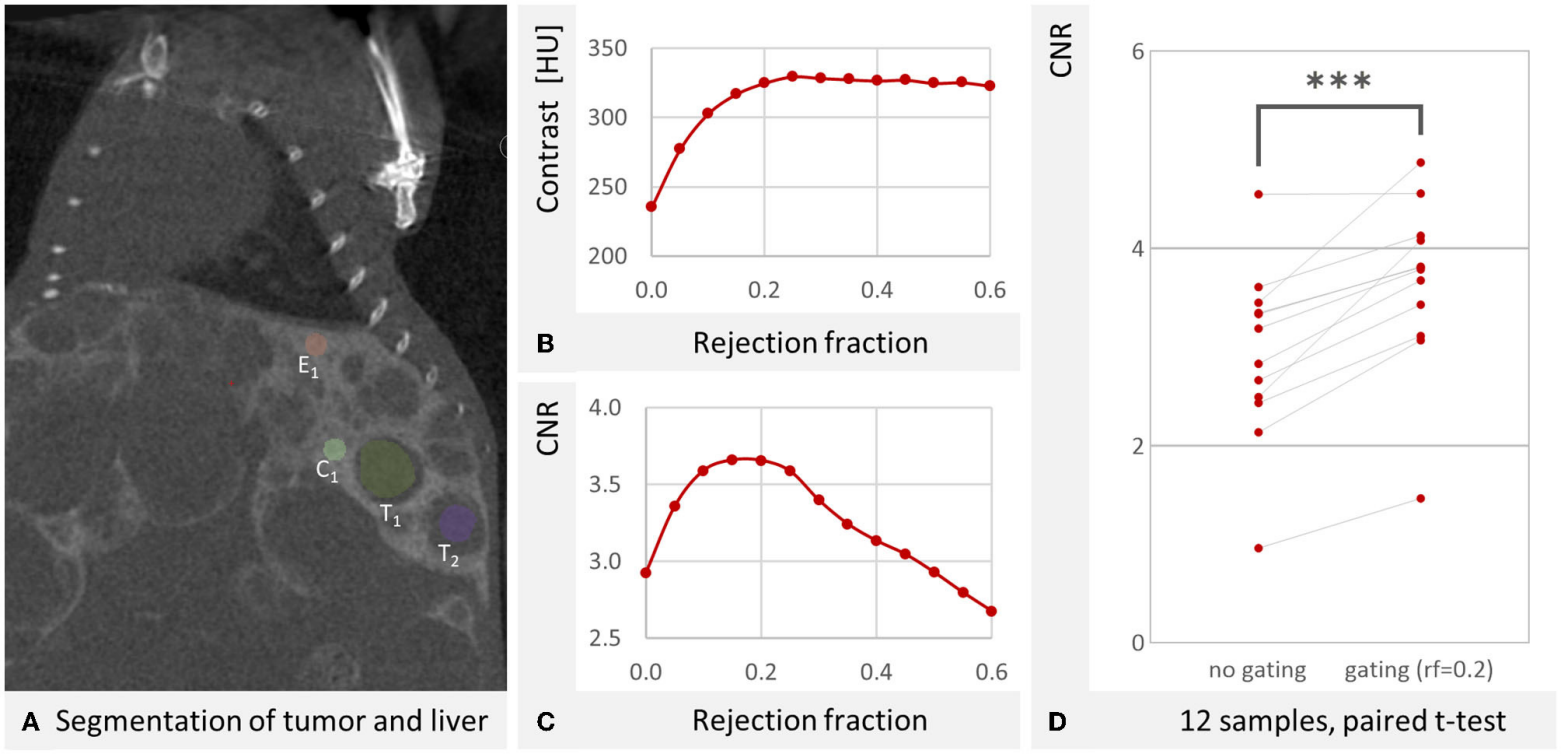
Contrast of liver vs. tumor tissue. **(A)** An exemplary coronal μCT slice through the liver. One of the spheres at the liver edge E_1_ and one of the spheres at the center C_1_ can be seen. The other measurement spheres are in other slices. Also visible is the segmentation of two tumors T_1_ and T_2_. The third segmented tumor is not visible in this slice. The artifact at the top of the image is due to the fact that this is also the edge of the volumetric image, where less data is available for reconstruction. W: 4,429 HU L: 670 HU **(B)** Averaged over all mouse samples, the contrast between liver and tumor increases with RF and reaches saturation at RF = 0.25. **(C)** Averaged over all mouse samples an optimum contrast to noise ratio of 3.659 was determined at RF = 0.15, similar to 3.657 at RF = 0.2. The noise was derived from a homogenous area of the mouse bed. **(D)** For the twelve reconstructions, the CNR is significantly (*p* < 0.001 as indicated by ***, paired *t*-test) improved by the gating (RF = 0.2).

The overall goal of this study is to improve liver tumor imaging. Therefore, we examined liver vs. tumor contrast-to-noise-ratio (CNR). The voxel noise was derived from a homogenous area in the mouse bed. Contrast and CNR were plotted over the rejection fraction in Figure 9. The contrast increases with RF and saturates at 25%. The optimum RF for tumor detectability in our scenario was found at 15% RF. This can be explained by increased noise at higher RFs due to the exclusion of too many projections. At a rejection fraction of 20%, our gating resulted in a significant increase of the CNR (*p* < 0.001, paired *t*-test, Figure 9).

## Discussion

We provided a method to improve μCT reconstructions, while maintaining a high throughput setting. Most notably is the ease of use and the possibility to apply the method to already existing scans since gated acquisition is not necessary and acquiring multiple projections from a single gantry angle is not a requirement.

### Prerequisites

An essential prerequisite of our method is the described anesthesia. We demonstrated in section 3.1 that in a four mice scan only about 21% of the projections are affected by respiratory motion due to the gasping breathing. This may change with the dosage of the anesthetics. While this was not analyzed systematically, induction of the required gasping breathing behavior may require a comparatively strong anesthesia. The advantages of the presented gating method (less wasted X-ray dosage and shorter anesthesia) must therefore be weighed against the disadvantages of the potentially more stressful anesthesia. However, in our experience, a somewhat stronger anesthesia is acceptable for the short μCT scanning duration of a few minutes and is also beneficial to reduce the risk that a mouse wakes up during the scan which is stressful for the animal and operator and typically results in repetition of the failed scan.

The demands on the computing power of the hardware are high for the presented method. An iterative reconstruction needs many computing steps. Our reconstruction requires a reasonable amount of time (~12 minutes per mouse on our sytem) because the calculation steps are parallelized using GPU-Accelerated Adjoint Algorithmic Differentiation (29). Further speed-ups are possible by using more and stronger GPUs. Furthermore, four reconstructions with full field of view are required per scan, i.e., one per mouse.

### Limitations and Future Work

While we evaluated a 4-mice-bed and compared the results to single-mouse scans, it would be interesting to also test 2- or 3-, or 5-mice-beds to find the best compromise between throughput and image quality.

We did not make a direct comparison between iterative reconstruction and filtered back projection (FBP). This would be interesting to better investigate the tradeoff between the expected better quality of iterative reconstruction and the significantly lower computational requirements of FBP. With FBP, one could also evaluate different approaches to compensate for the missing projections. A full reconstruction with FBP takes about as long as a single pass of iterative reconstruction. We used 30 iterations each for this study. Future work could vary this number and investigate the impact on quality and computation time.

A previously described method for single-mouse scans also places an ROI on the diaphragm for intrinsic retrospective gating and discards motion affected projections (13). Because that method uses a Feldkamp type volumetric reconstruction, missing angular projections were substituted with projections from other rotations or interpolated from neighboring projections. However, the authors concluded that in general, dose usage is better in prospective methods, because all acquired projections contribute to image reconstruction (13). This was more recently addressed by means of a model-based reconstruction in combination with motion compensating vector fields (32) or motion estimation using a non-rigid registration method (21). In both cases, the motion affected projections are reshaped so that they can be included in the reconstruction to preferably use all acquired data. The method we presented might also further increase its dose efficiency with similar additions.

For further improvements of the image quality, different scan protocols could be investigated, e.g., with different voltages, exposure times, or angle steps. Moreover, a continuous rotation scan protocol could be used. Because the X-ray source radiates continuously, less dose would be wasted for stop and go, at the cost of some motion blur caused by the rotation.

The productivity of our method could be improved by reducing the required user interventions. It would be desirable if the software itself were able to determine the ROI, which has already been described (28). Work in progress is aiming to automatically detect and segment the lung toward this end. Instead of presetting a fixed RF before reconstruction, the algorithm itself could find an optimal RF for each scan.

## Conclusion

We can conclude that 4-mouse gating is possible with only a small loss of data (rejected projections) compared to single-mouse gating. This means that gating and high throughput are compatible. Our gating approach improves image quality i.e., the detectability of tumors in a tumor bearing liver for a 4-mouse scan without substantial compromise to dose efficiency compared to a single-mouse scan.

## Data Availability Statement

The datasets presented in this article are not readily available because they are property of Roche Diagnostics. Requests to access the datasets should be directed to FG, fgremse@ukaachen.de.

## Ethics Statement

The animal study was reviewed and approved by the Government of Upper Bavaria (Regierung von Oberbayern).

## Author Contributions

MT performed the gated reconstructions, analyzed the data, and wrote the original manuscript. SR and FG helped with the statistical analysis. AH, KL, and FG implemented the iterative reconstruction software. FO and TP planned and conducted the animal experiments. SR, KL, AH, FK, FO, TP, and FG reviewed the article. FG supervised the study and revised the article. All authors contributed to the article and approved the submitted version.

## Funding

The authors would like to thank the Federal Government of North-Rhine Westphalia, and the European Union (EFRE), the German Research Foundation (GR 5027/2-1 and CRC1382 project ID 403224013 - SFB 1382, project Q1) for funding.

## Conflict of Interest

FG is the owner of Gremse-IT GmbH, a spin-out of the RWTH Aachen University, which commercializes software for biomedical image analysis. TP and FO are employees of Roche Diagnostics GmbH. The remaining authors declare that the research was conducted in the absence of any commercial or financial relationships that could be construed as a potential conflict of interest.

## Publisher's Note

All claims expressed in this article are solely those of the authors and do not necessarily represent those of their affiliated organizations, or those of the publisher, the editors and the reviewers. Any product that may be evaluated in this article, or claim that may be made by its manufacturer, is not guaranteed or endorsed by the publisher.
